# O Índice de Inflamação Imune Sistêmica é um Novo Marcador na Previsão da Presença e Gravidade aa Ectasia Coronariana Isolada

**DOI:** 10.36660/abc.20220056

**Published:** 2022-12-20

**Authors:** Ferhat Dindas, Emin Koyun, Erdem Turkyilmaz, Ozge Ozcan Abacioglu, Arafat Yildirim, Anil Sahin, Baris Dindar, Mustafa Dogdus, Ozkan Candan

**Affiliations:** 1 Usak University Training and Research Hospital Department of Cardiology Usak Turquia Usak University , Training and Research Hospital , Department of Cardiology , Usak – Turquia; 2 Sivas Cumhuriyet University Department of Cardiology Sivas Turquia Sivas Cumhuriyet University , Department of Cardiology , Sivas – Turquia; 3 University of Health Sciences Adana Health Practice and Research Center Adana Turquia University of Health Sciences , Adana Health Practice and Research Center , Adana – Turquia

**Keywords:** Doença Arterial Coronariana/complicações, Dilatação Patológica, Biomarcadores, Inflamação Imune Sistêmica

## Abstract

**Fundamento:**

A patologia subjacente da ectasia da artéria coronária (EC) isolada não foi totalmente elucidada.

**Objetivo:**

Nosso objetivo foi examinar a relação entre o índice de inflamação imune sistêmica (Sıı), que corresponde à multiplicação da razão neutrófilos-linfócitos (RNL) e as contagens de plaquetas, e EC isolada.

**Método:**

A população do estudo retrospectivo incluiu 200 pacientes com EC isolada, 200 consecutivos com doença arterial coronariana obstrutiva e 200 consecutivos com angiografia coronária normal. Um valor de p bicaudal <0,05 foi considerado significativo.

**Resultados:**

Sıı, RNL, razão plaqueta-linfócito (RPL) e razão monócito-colesterol de lipoproteína de alta densidade (MHR) foram significativamente maiores no grupo EC em comparação com os outros grupos (todos p<0,001). Na análise multivariada, Sıı (p<0,001, OR = 1,005, IC 95% =1,004-1,005) foi considerado um preditor independente de EC isolada. Na análise da curva Receiver Operating Characteristic (ROC), Sıı teve uma área sob a curva maior em comparação com RNL, RPL e MHR. O valor de Sıı >517,35 tem 79% de sensibilidade, 76% de especificidade para a predição do EC [AUC: 0,832, (p<0,001)]. Sıı teve correlação significativa com o número de artérias coronárias ectásicas e classificação de Markis (r: 0,214 p=0,002; r:-0,195, p=0,006, respectivamente).

**Conclusão:**

Até onde sabemos, este é o primeiro estudo em que Sıı foi significativamente associado à presença isolada de EC e gravidade anatômica.

## Introdução

A ectasia da artéria coronária (EC) é definida como o aumento de um ou mais segmentos da artéria coronária epicárdica excedendo 1,5 vezes o segmento adjacente. ^1^ A EC é um achado patológico angiograficamente não oclusivo nas artérias coronárias. ^2^ A EC é uma importante entidade clínica uma vez que desenvolve doença arterial coronariana (DAC) oclusiva em 34%. ^3^ Muitos componentes celulares e moleculares complexos estão envolvidos na cascata patobiológica da EC. Estudos recentes comparando EC com DAC e aneurismas de artéria coronária constataram que a patologia baseada na inflamação é predominante. ^4 - 6^

Biomarcadores inflamatórios de procedência hematológica, especialmente a razão neutrófilo-linfócito (RNL) e a razão plaqueta-linfócito (RPL), ganharam recentemente uma reputação de predizer eventos adversos cardiovasculares. ^7^ Outro índice inflamatório e relacionado à aterosclerose é à razão monócito-colesterol de lipoproteína de alta densidade (MHR). ^8^ Hu et al. desenvolveram um marcador racional inovador e previsível chamado índice inflamatório imunológico sistêmico (Sıı) com base em um estudo de coorte ambispectivo. ^9^ O Sıı, que corresponde à multiplicação do RNL e do número de plaquetas (PN/L), é um parâmetro que mostra a resposta imune sistêmica e local. ^9^ O Sıı foi associado a um evento cardiovascular adverso importante em pacientes idosos com infarto agudo do miocárdio. ^10^ Estudos recentes encontraram uma correlação entre Sıı e a gravidade da doença arterial coronariana. ^11 , 12^ O Sıı inclui uma combinação de três células inflamatórias hematológicas, propriedade que o torna mais valioso do que outros parâmetros inflamatórios em estudos atuais.

Assim, nosso objetivo foi investigar a possível relação entre EC isolada e um novo parâmetro de inflamação, Sıı, em pacientes com angina pectoris estável ou instável submetidos à angiografia coronária (AGC).

## Métodos

### População do estudo e ética

Incluímos inicialmente 252 pacientes com EC retrospectivamente que realizaram AGC com pré-diagnóstico de DAC estável e instável entre janeiro de 2011 e dezembro de 2019. Para o diagnóstico, tratamento ou exclusão de DAC, a AGC foi realizada na presença de dor torácica típica e acompanhada de um ou mais exames, como testes de esteira positivos, cintilografia de perfusão miocárdica anormal e angiotomografia coronária anormal.

Pacientes com história de intervenção coronária percutânea e cirurgia de revascularização do miocárdio, presença de infarto agudo do miocárdio com supradesnivelamento ou não do segmento ST, doenças infecciosas, hematológicas, inflamatórias, insuficiência renal avançada (taxa de filtração glomerular estimada < 30) e hepática (razão normalizada internacional prolongada (> 1,5) com aumento do nível sérico de bilirrubina total, alanina aminotransferase e aspartato aminotransferase) e malignidade diagnosticada foram excluídos do estudo. Os 200 pacientes consecutivos restantes com EC isolada, os 200 pacientes consecutivos com DAC obstrutiva sem ectasia coronariana e os 200 pacientes consecutivos com artéria coronária angiograficamente normal foram pareados para formar três grupos. A hiperlipidemia foi definida como colesterol total (CT) > 200 mg/dL ou colesterol de lipoproteína de baixa densidade (LDL-c) > 160 mg/dL ou uso de terapia com estatina. Diabetes mellitus foi definido como glicemia de jejum > 126 mg/dL ou uso de qualquer agente antidiabético. A história familiar positiva foi definida por um parente de primeiro grau antes dos 55 anos em homens e 65 em mulheres com DAC ou morte súbita. Hipertensão foi definida como pressão arterial sistólica ≥ 140 mmHg e/ou pressão arterial diastólica ≥ 90 mmHg ou qualquer uso de medicação anti-hipertensiva relatado. Tabagismo foi definido como tabagismo por mais de um ano-maço. O comitê de ética local aprovou o protocolo do estudo seguindo a Declaração de Helsinque (12/9/2021). Devido à natureza retrospectiva do estudo, o consentimento do paciente foi dispensado pelo comitê de ética.

### Avaliação AGC

A AGC de todos os pacientes incluídos no estudo foi realizada pela técnica de Judkins e cateteres 6-French da artéria femoral ou radial. Nossa clínica de cardiologia registrou todas as imagens angiográficas no sistema digital Philips Allura Xper Percutaneous Coronary Intervention. O contraste de iopromida (Omnipaque; GE Healthcare) foi usado em todos os pacientes do estudo. Foram avaliadas as imagens angiográficas digitais registradas em pelo menos quatro cineprojeções para o sistema coronariano esquerdo e pelo menos duas cineprojeções para o sistema coronariano direito. As imagens angiográficas foram avaliadas por dois cardiologistas cegos para os detalhes do estudo. A EC foi definida como o aumento de qualquer segmento de qualquer artéria coronária principal para pelo menos 1,5 vezes o diâmetro do segmento adjacente sem lesão que cause estenose maior que 50%. O grupo EC isolada foi dividido em quatro tipos de acordo com a classificação de Markis: ectasia difusa em duas ou três artérias coronárias Tipo I; ectasia difusa em uma artéria coronária e ectasia localizada em outras artérias Tipo II; ectasia difusa em apenas uma artéria coronária Tipo III; lesões ectásicas localizadas e segmentares Tipo IV. ^13^ O número de artérias coronárias ectásicas foi obtido avaliando-se apenas as artérias coronárias principais. TCE, ADAE, ACXE, CD). O grupo DAC obstrutiva foi definido como estenose >50% em uma ou mais artérias coronárias principais e sem ectasia em nenhuma artéria coronária. O grupo de artéria coronária normal foi definido como a ausência de qualquer DAC causando irregularidade do lúmen visual no AGC. ectasia difusa em uma artéria coronária e ectasia localizada em outras artérias Tipo II; ectasia difusa em apenas uma artéria coronária Tipo III; lesões ectásicas localizadas e segmentares Tipo IV.13 O número de artérias coronárias ectásicas foi obtido avaliando-se apenas as artérias coronárias principais. (TCE, ADAE, ACXE, CD). O grupo DAC obstrutiva foi definido como estenose >50% em uma ou mais artérias coronárias principais e sem ectasia em nenhuma artéria coronária. O grupo de artéria coronária normal foi definido como a ausência de qualquer DAC causando irregularidade do lúmen visual na AGC.

### Medições laboratoriais

Amostras de sangue venoso periférico foram coletadas dos pacientes antes da AGC no momento da admissão. Parâmetros de células sanguíneas completas, amostras de sangue foram coletadas em tubos de 3,0 ml contendo 5,40 mg de ácido etilenodiamino tetraacético (EDTA) seco e analisadas usando um contador automático de células sanguíneas (Beckman Coulter, EUA). Os níveis séricos de triglicerídeos (TG), lipoproteína de alta densidade (HDL-c) e CT foram quantificados com métodos enzimáticos padrão (Abbot GmbH Co, Alemanha) com um analisador totalmente automatizado (Abbott Architect c16000) com reagentes originais. A concentração de LDL-c foi determinada de acordo com o método de Friedewald. A concentração de proteína C-reativa (PCR) foi medida com um analisador químico automatizado. RNL, RPL e MHR foram calculadas como contagem absoluta de neutrófilos/contagem absoluta de linfócitos, contagem absoluta de plaquetas/contagem absoluta de linfócitos e contagem absoluta de monócitos/nível absoluto de HDL-c, respectivamente. Sıı foi calculado como contagem absoluta de neutrófilos x contagem de plaquetas/contagem absoluta de linfócitos. ^14^

### Análise estatística

Todos os dados do estudo foram analisados com o software SPSS (IBM SPSS Statistics for Windows, Versão 21.0. Armonk, NY, EUA, IBM Corp.). O teste de Kolmogorov-Smirnov foi realizado para confirmar se as variáveis apresentavam distribuição normal. As variáveis contínuas foram indicadas como mediana e intervalo interquartil (percentil 25-75), e as variáveis categóricas foram indicadas como frequências e porcentagens. O teste H de Kruskal-Wallis foi utilizado na comparação de três grupos independentes para analisar as variáveis que não se enquadravam na distribuição normal. O teste post hoc de Dunn-Bonferroni foi usado para comparações pareadas. As variáveis categóricas foram analisadas por meio do teste qui-quadrado apropriado. A correlação entre as variáveis foi avaliada por meio do teste de correlação de Spearman’s Rank. A análise da curva Receiver Operating Characteristic (ROC) foi usada para determinar o papel preditivo das variáveis.

A análise de regressão logística multivariada foi empregada para determinar as variáveis independentes para EC isolado. Para a regressão multivariada, as variáveis com valor de p < 0,1 na análise de regressão logística univariada foram identificadas como potenciais marcadores de risco e incluídas no modelo completo. O teste de Hosmer-Lemeshow verificou um ajuste adequado para o modelo de regressão. Plaquetas, neutrófilos e linfócitos não foram incluídos nos modelos de regressão para evitar multicolinearidade. As razões de possibilidades (ORs) foram apresentadas com 95% de seus respectivos intervalos de confiança (IC). Um valor de p bicaudal <0,05 foi considerado significativo.

## Resultados

**Figura 1:**
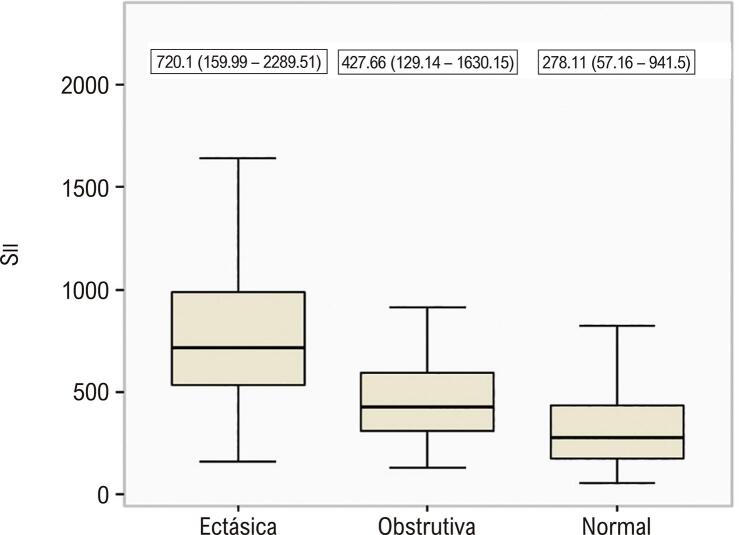
Valores Sıı de grupos.

**Figura 2:**
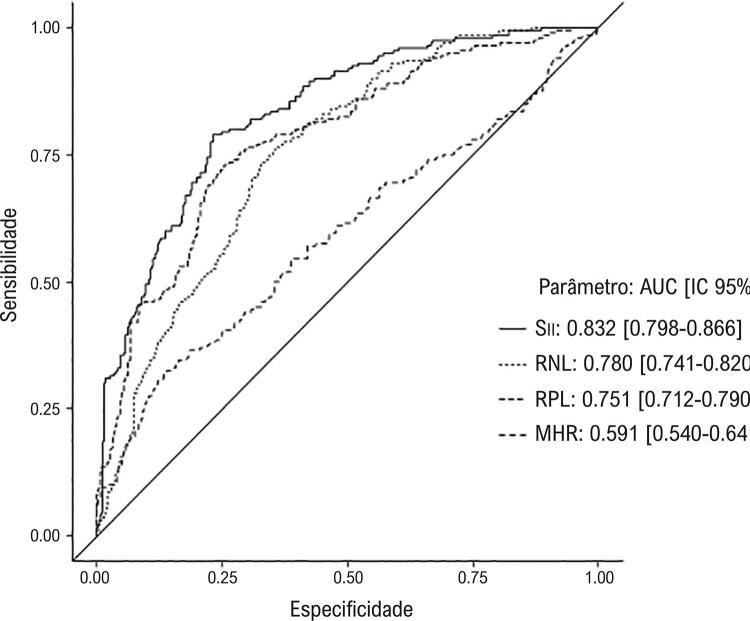
Análise da curva ROC de Sıı, RNL, RPL e MHR para prever EC isolada. IC: intervalo de confiança; MHR: razão monócito-colesterol de lipoproteína de alta densidade; RPL: proporção de plaquetas para linfócitos; RNL: razão de neutrófilos para linfócitos; Sıı: índice de imuno inflamação sistêmica.

**Figura 3:**
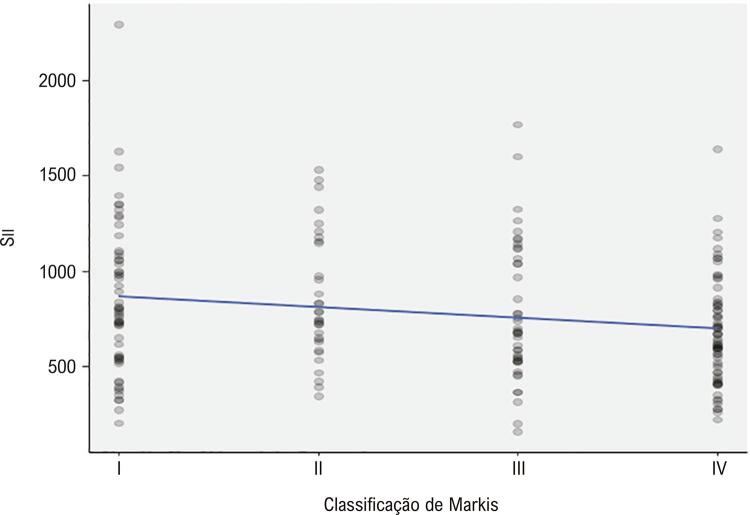
Correlação de Sıı com a classificação de Markis.

**Figura 4:**
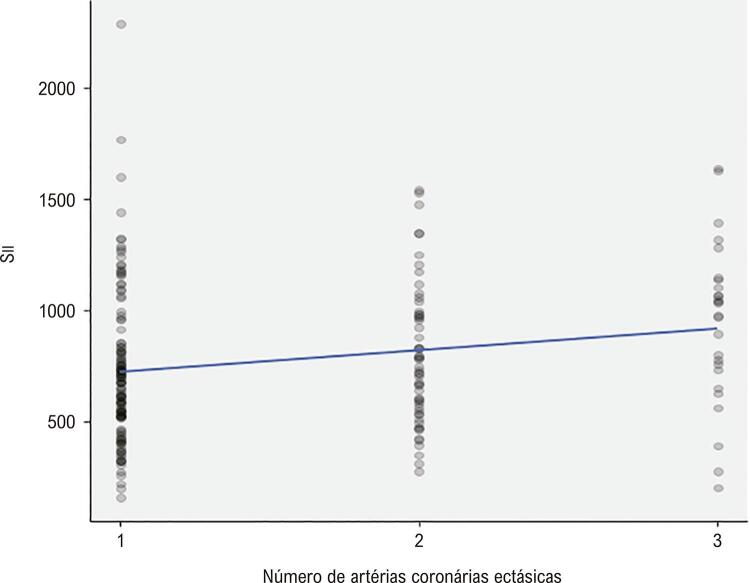
Correlação de Sıı com o número da artéria coronária ectásica.

As características clínicas e angiográficas basais da população estudada são apresentadas na Tabela 1 . Não houve diferença entre os três grupos quanto à idade, sexo, FEVE, dislipidemia, história familiar, hipertensão e medicações prévias. O tabagismo foi maior no grupo com artéria coronária ectásica, enquanto a frequência de diabetes mellitus foi mais prevalente no grupo com artéria coronária obstrutiva. A frequência de artérias coronárias com ectasia em nosso estudo foi a seguinte; artéria coronária direita (CD) 62,5%, artéria descendente anterior esquerda (ADAE) 52%, artéria circunflexa (CX) 38,5%, tronco da artéria coronária esquerda (TCE) 6,5%. Distribuição de acordo com a gravidade da EC: Markis Tipo I 28,5%, Markis Tipo II 16,0%, Markis Tipo III 21,5%, Markis Tipo IV 34,0%. Das quatro localizações de artérias coronárias que consistem em TCE, ADAE, CX e CD, EC foi de 13% em três artérias coronárias, 27,5% em duas artérias coronárias e 59,5% em uma artéria coronária. Os achados laboratoriais dos grupos de estudo são apresentados na Tabela 2 . Os pacientes com EC isolada apresentaram leucócitos (leucócitos), contagem de neutrófilos, contagem de plaquetas, MHR, RPL e RNL significativamente maiores. Conforme apresentado na Figura 1 , Sıı foi significativamente maior no grupo EC isolada do que nos demais grupos. Por outro lado, o grupo EC isolada apresentou níveis de ALT e contagens de linfócitos significativamente mais baixos. Os níveis de PCR foram significativamente maiores no grupo de artéria coronária obstrutiva do que em outros grupos. Tabagismo, diabetes mellitus, TG, ALT, PCR, MHR, RPL, RNL e Sıı foram avaliados por análise de regressão para detectar os determinantes univariados de EC isolada. Na análise de regressão logística multivariada, tabagismo, diabetes mellitus, MHR, RPL, RNL, e Sıı foram preditores independentes e significativos de EC isolada ( Tabela 3 ). Na análise da curva ROC para estimar o EC isolada, Sıı apresenta a maior AUC com valor de corte de 517,35 ( Figura 2 ). Sıı correlacionou-se significativamente negativamente com a classificação de Markis ( Figura 3 ). Além disso, Sıı correlacionou-se significativamente com o número de artérias coronárias ectásicas ( Figura 4 ).

**Tabela 1:** Comparação das características clínicas e angiográficas basais dos grupos

Variáveis	Ectasiado grupo de artérias coronárias (n=200)	Obstrutivo coronário grupo de artérias (n=200)	Grupo de artéria coronária normal (n=200)	p
Anos de idade	58 (52 - 65)	59 (52 - 66)	59 (52 - 63)	0,348
FEVE, (%)	52 (52 - 57)	52 (52 - 55)	52 (51 - 55)	0,918
Sexo masculino, n (%)	143 (71,5)	151 (75,5)	139 (69,5)	0,395
Dislipidemia, n (%)	64 (32,0)	77 (38,5)	61 (30,5)	0,198
História familiar, n (%)	82 (41,0)	88 (44,0)	76 (38,0)	0,475
Fumante, n (%)	86 (43,0)	70 (35,2)	48 (24,0)	<0,001
Hipertensão, n (%)	110 (55,0)	103 (51,5)	97 (48,5)	0,428
Diabetes mellitus, n (%)	57 (28,5)	89 (44,5)	65 (32,8)	0,002
**Medicamentos, n(%)**				
iECA-BRA	88 (44,0)	97 (48,5)	84 (42,0)	0,408
BCC	45 (22,5)	54 (27,0)	43 (21,5)	0,387
APT	36 (18,0)	38 (19,0)	32 (16,0)	0,726
BB	61 (30,5)	62 (31,0)	47 (23,5)	0,177
Terapia com estatina	71 (35,5)	76 (38,0)	62 (31,0)	0,330
**Artéria coronária, n(%)**				
TCE	13 (6,5)			
ADAE	104 (52,0)			
CX	77 (38,5)			
ACD	125 (62,5)			
**Classificação de Markis, n(%)**				
Tipo I	57 (28,5)			
Tipo II	32 (16,0)			
Tipo III	43 (21,5)			
Tipo IV	68 (34,0)			
**Número de artérias coronárias ectásicas, n(%)**				
1	119 (59,5)			
2	55 (27,5)			
3	26 (13,0)			

*iECA: inibidor da enzima conversora de angiotensina; APT: terapia antiplaquetária; BRA: bloqueadores do receptor da angiotensina 2; BB: betabloqueador; BCC: bloqueador dos canais de cálcio; CX: artéria circunflexa; FEVE: fração de ejeção do ventrículo esquerdo; ADAE: artéria descendente anterior esquerda; TCE: tronco de coronária esquerda; ACD: artéria coronária direita.*

**Tabela 2:** Comparação dos parâmetros laboratoriais dos grupos de estudo

Variáveis	Grupo de artérias coronárias ectásicas (n=200)	Coronária obstrutiva grupo de artérias (n=200)	Grupo de artéria coronária normal (n=200)	p
BUN, mg/dL	17 (13 - 21)	16 (13 - 21)	16 (13 - 19,6)	0,535
Creatinina, mg/dL	0,9 (0,7 - 1,3)	0,97 (0,81 - 1,18)	0,95 (0,80 - 1,18)	0,600
PCR, mg/L	3,4 (1,93 - 7,58) ^ab^	2,25 (1,3 - 9,30)a	4,82 (3,0 – 6,0) ^b^	**0,001**
ALT, IU/L	20 (15 - 30) ^a^	24 (18 - 33,8) ^b^	23,5 (17 - 32) ^b^	**0,005**
Hemoglobina, g/dL	14,8 (13,4 - 15,6)	14,2 (13,2 - 15,5)	14,3 (13,1 - 15,4)	0,190
RDW, %	11,85 (11,5 - 12,6)	11,9 (11,5 - 12,9)	12,1 (11,5 - 13,1)	0,212
WBC, x109 /L	9,16 (7,83 - 10,86) ^a^	8,72 (7,1 – 10,8) ^a^	7,79 (6,64 – 9,25) ^b^	**<0,001**
Plaqueta, x109 /L	279 (235 – 321,7) ^a^	222,5 (192,3 – 254,5) ^b^	226 (195 - 261) ^b^	**<0,001**
VCM (fL)	87,05 (80,5 – 90,1)	87,2 (84,9 - 90,1)	87,1 (80,4 - 90,1)	0,552
VPM (fL)	7,6 (7,1 - 8,3)	7,4 (7,0 - 8,2)	7,7 (6,9 - 8,3)	0,327
Neutrófilo, x109 /L	5,7 (4,63 - 6,99) ^a^	4,97 (3,82 – 6,48) ^b^	3,58 (2,19 - 5,05) ^c^	**<0,001**
Monócito, x109 /L	0,53 (0,41 - 0,73) ^a^	0,49 (0,38 - 0,61) ^b^	0,51 (0,39 - 0,61) ^b^	**0,003**
Linfócito, x109/L	2,21 (1,75 - 2,77) ^a^	2,69 (2,04 – 3,22) ^b^	2,7 (2,17 - 3,40) ^b^	**<0,001**
TG mg/dL	126 (91 - 182)	141 (103 - 200,7)	130 (94 - 192)	0,081
CT, mg/dL	175 (143 - 203)	185 (157 – 214,3)	180 (150 - 208)	0,222
LDL-c, mg/dL	104 (77,7 - 125)	104,3 (78 - 134)	98 (75 - 123)	0,529
HDL-c, mg/dL	37 (31 - 45)	38 (32,2 - 46)	36 (31 - 44)	0,256
MHR	0,01 (0,01 - 0,02) ^a^	0,01 (0,01 - 0,02) ^b^	0,01 (0,1 - 0,02) ^a,b^	**<0,001**
RNL	2,59 (1,94 - 3,40) ^a^	1,83 (1,41 - 2,76) ^b^	1,3 (0,74 - 1,98)c	**<0,001**
RPL	124,2 (101,3 - 163,0) ^a^	82,6 (66,4 - 106,8) ^b^	85,4 (60,9 – 103,0) ^b^	**<0,001**
Sıı	720,1 (532,9 - 991,8) ^a^	427,6 (307,5 – 597,8) ^b^	278,1 (172,0 - 436,1) ^c^	**<0,001**

*ALT: alanina aminotransferase; BUN: azoto ureico no sangue; PCR: proteína C reativa; HDL-c: colesterol de lipoproteína de alta densidade; VCM: volume corpuscular médio; MHR: razão monócito-colesterol de lipoproteína de alta densidade; RNL: razão de neutrófilos para linfócitos; VPM: volume plaquetário médio; LDL-c: colesterol de lipoproteína de baixa densidade; RPL: proporção de plaquetas para linfócitos; RDW: largura de distribuição das células vermelhas; Sıı: índice de imuno-inflamação sistêmica; CT: colesterol total; TG: triglicerídeo; WBC: glóbulo branco. As variáveis são expressas como mediana (25ª–75ª). Letras semelhantes na mesma linha mostram a semelhança entre os grupos; letras diferentes mostram a diferença entre os grupos.*

**Tabela 3:** Preditores de EC isolada por análise de regressão logística multivariada

Variáveis	Análise Univariada		Análise multivariada	

	p	OU (IC 95%)	p	OR ajustado (IC 95%)
Fumar	**0,001**	1,796 (1,262-2,558)	**<0,001**	1.824 (1.189-2.803)
Diabetes mellitus	**0,014**	0,632 (0,435-0,908)	**<0,001**	0,644 (0,412-0,998)
TG	0,256	0,999 (0,997-1,001)		
ALT	0,320	0,996 (0,986-1,004)		
PCR	0,233	1,016 (0,99-1,042)		
MHR	**<0,001**	1.062 (1.036-1.089)	**<0,001**	1,039 (1,007-1,073)
RPL	**<0,001**	1.026 (1.021-1.032)	**<0,001**	1.027 (1.022-1.033)
RNL	**<0,001**	2,02 (1,717-2,4)	**<0,001**	2.024 (1.708-2.423)
Sıı	**<0,001**	1,005 (1,004-1,005)	**<0,001**	1,004 (1,004-1,005)

*EC: ectásia da artéria coronária; IC: intervalo de confiança; OR: razão de chances; p: valor p. TG: triglicerídeo; ALT: alanina aminotransferase; PCR: proteína C reativa; MHR: razão monócito-colesterol de lipoproteína de alta densidade; RPL: proporção de plaquetas para linfócitos; RNL: razão de neutrófilos para linfócitos; Sıı: índice de imuno-inflamação sistêmica.*

## Discussão

Nosso objetivo foi investigar a relação entre EC isolada e Sıı, um novo marcador incluindo contagem de neutrófilos, plaquetas e linfócitos. Nosso estudo mostrou que pacientes com EC isolada apresentaram valores de Sıı significativamente maiores do que pacientes com artérias coronárias obstrutivas e normais. Em particular, a gravidade da EC isolada e sua extensão nas artérias coronárias estão independentemente associadas a um aumento de Sıı, refletindo a ativação inflamatória.

Segmentos coronarianos dilatados, preenchimento anterógrado de corante coronariano prejudicado, deposição localizada de corante com estase e fenômeno de vazamento retrógrado são as principais características angiográficas coronarianas da EC. ^15^ A incidência relatada de EC varia de 0,3% a 10%, dependendo do número de pacientes submetidos à ACG. ^16 , 17^ Em um estudo transversal, verificou-se que a frequência de eventos cardiovasculares aumentou na presença de EC acompanhando DAC. ^18^ Em outro estudo recente, a presença de EC afeta a mortalidade cardiovascular futura em pacientes com infarto agudo do miocárdio. ^19^ O aumento da detecção da ectasia das artérias coronárias, mostrando sua relação com a mortalidade cardiovascular, é um dos fatores predisponentes do infarto agudo do miocárdio e associado à isquemia microvascular na ausência de lesão obstrutiva concomitante, aumentou sua importância clínica na última década. ^15 , 18 - 20^ A artéria coronária mais acometida pela ectasia é a CD, seguida pela ADAE e CX, e a coronária menos acometida artéria é o TCE. ^6^ A EC tem características demográficas de homens mais jovens em comparação com pacientes com DAC obstrutiva. ^21^ Em nosso estudo, não houve diferença demográfica significativa entre todos os grupos, e os pacientes com EC isolada foram semelhantes aos pacientes com DAC obstrutiva quanto ao evento cardiovascular fatores de risco. Em consonância com a literatura, em nosso estudo, houve relação linear entre tabagismo, hipertensão e hiperlipidemia em pacientes com EC, enquanto uma relação inversa foi encontrada com diabetes mellitus. ^2^ Essa relação inversa se deve ao diabetes mellitus prejudicar a dilatação arterial compensatória ao promover processo aterosclerótico ao remodelamento negativo da parede arterial. ^4^ O título do processo aterosclerótico na patogênese da EC, que é considerado um tipo de DAC, permanece incerto. ^5^ Com base nos resultados dessas publicações anteriores comparando EC e aneurismas de artéria coronária, a atividade inflamatória é mais evidente na EC. ^22^ Por essas razões, embora a patogênese da EC não tenha sido totalmente elucidada, mecanismos inflamatórios e outros prováveis, ao invés do processo aterosclerótico, tornaram-se tópicos mais destacados. ^4 , 5^

Existem vários estudos sobre a relação entre EC e inflamação. Demir et al., ^23^ mostraram que níveis séricos elevados de PCR sensível, ácido úrico e níveis séricos mais baixos de bilirrubina, que são indicadores da resposta inflamatória, estão associados à presença de ectasia. ^23^ Turan et al., ^24^ ilustraram uma correlação entre os níveis de endocan indicando disfunção endotelial e processo inflamatório e EC. ^24^ Finkelstein et al., ^25^ mostraram que o desequilíbrio da metaloproteinase da matriz circulatória, que está correlacionado com marcadores inflamatórios, está associado à formação de EC. ^25^ Jun Li et al., ^5^ ilustraram que a EC estava associada à contagem de interleucina-6, leucócitos, neutrófilos e monócitos e afirmaram que o processo inflamatório crônico está envolvido na EC. ^5^ Ashraf et al., ^26^ encontraram uma correlação entre o hormônio do adipócito visfatina, que desempenha um papel fundamental no retardo da apoptose de neutrófilos e na gravidade da EC. ^26^ Adiloglu et al. ^27^ mostraram que as moléculas de adesão da superfície celular, que são necessárias para o início da inflamação na EC, são mais difundidas no endotélio. ^27^ Além disso, vários estudos descobriram que RNL, RPL e MHR, que são parâmetros inflamatórios tradicionais, são preditores independentes de EC e estão correlacionados com o número e gravidade dos vasos ectásicos. ^28 - 30^ Consistentemente com isso, RNL, RPL e MHR foram preditores independentes de EC isolada em nosso estudo. Embora a PCR tenha sido maior no grupo EC isolada do que nos outros grupos, não conseguimos detectá-la como preditor independente. Esse resultado pode ser devido à ausência de valores elevados de PCR sensível em nosso estudo.

Sıı é um novo marcador inflamatório hematológico que reúne neutrófilos, plaquetas e linfócitos, refletindo inflamação e equilíbrio imunológico. Sıı foi definido pela primeira vez no campo da oncologia. O excesso de índice inflamatório sistêmico tem sido associado à contagem de células cancerígenas circulantes, prognóstico ruim e menor tempo de sobrevida em pacientes com câncer. ^9 , 14^ Em estudos de câncer recentes, o papel prognóstico de Sıı foi considerado mais potente do que RNL e RPL. ^31^ Além disso, Sıı como marcador prognóstico tem sido demonstrado em pacientes com doenças cardiovasculares. ^10 - 12^ Há evidências crescentes de que neutrófilos e produtos derivados de neutrófilos participam da EC. ^32^ No processo patobiológico da EC, há um aumento de moléculas adesivas na superfície celular. ^26^ Subsequentemente, a degranulação da matriz extracelular ocorre devido às moléculas de adesina que proporcionam a transmigração de monócitos e espalham a serina proteinase derivada de neutrófilos. ^26 , 33 , 34^ Estudos anteriores relataram que plaquetas, quimiocinas e citocinas aumentam a migração de células progenitoras para a área inflamatória que se desenvolve após a lesão endotelial. ^5 , 33^ As plaquetas desempenham papéis importantes na inflamação e na trombose. Sabe-se que a agregação plaquetária ativada está entre as principais causas de complicações cardiovasculares. ^35^ Recentemente, o marcador de ativação plaquetária, volume plaquetário médio, demonstrou ter valor prognóstico em eventos cardíacos adversos maiores em pacientes com EC isolada. ^36^ Como resultado da publicação anterior, níveis plasmáticos de P-selectina e fator-4 plaquetário, que se acredita aumentarem a ativação plaquetária em pacientes com EC isolada, foram maiores do que no grupo controle. ^37^ Em contraste com neutrófilos e plaquetas, a função de modulação imune dos linfócitos na inflamação é explicada pelo aumento da apoptose de linfócitos, regulação negativa da proliferação e diferenciação de linfócitos e diminuição da contagem de linfócitos. ^28^ Além disso, sabe-se que as leucocitoses neutrofílicas e linfopênicas são marcadores de mau prognóstico de várias doenças cardiovasculares principais. ^28^ À luz dos estudos relatados, levantamos a hipótese de que o nível de Sıı, que é conhecido por estar associado à inflamação e ativação do sistema imunológico, pode prever pacientes com EC. As análises da curva ROC em nosso estudo mostraram que Sıı teve uma AUC maior em comparação com RNL, RPL e MHR; isso nos levou a concluir que Sıı prediz pacientes com EC isolados melhor do que RNL, RPL e MHR. Além disso, Sıı surgiu como um preditor independente de EC isolado nos modelos de regressão multivariada. No entanto, houve uma correlação impotente entre Sıı e a gravidade anatômica do EC isolado. De acordo com esses resultados, dentre os índices hematológicos convencionais que podem ser obtidos de forma não invasiva a partir dos resultados do hemograma completo, o Sıı é mais aplicável na predição de pacientes com EC isolada. Além disso, Sıı pode ser um marcador interessante que merece investigação em diversas patologias cardíacas.

Existem algumas limitações em nosso estudo que devem ser mencionadas. É um estudo retrospectivo de pequeno tamanho amostral unicêntrico; os resultados carecem de acompanhamento a longo prazo e medidas de resultados. Além disso, o estudo não incluiu acompanhamento de longo prazo e medidas de resultados que demonstrem eficácia prognóstica. A razão PCR/albumina, outro importante parâmetro inflamatório, não foi avaliada devido à falta de valor da albumina nos resultados laboratoriais antes da AGC. Além disso, embora as avaliações angiográficas dos pacientes tenham sido determinadas por dois cardiologistas cegos para os dados do estudo, a possibilidade de viés não pode ser completamente excluída porque a variabilidade inter ou intra-observador não foi calculada. Embora o estudo inclua dados sobre o número de artérias coronárias ectásicas e classificação de Markis, Sıı não reflete diretamente a importância anatômica e inflamatória das EC isoladas, uma vez que não foram utilizadas técnicas para investigar vasculites imunes sistêmicas na etiologia.

## Conclusão

Em conclusão, a EC está associada ao aumento de Sıı e sugere a presença de processos inflamatórios em pacientes com EC isolada. Acreditamos que Sıı pode ser um índice mais eficaz do que outros parâmetros inflamatórios hematológicos na diferenciação de pacientes isolados de EC que precisarão de estratégias terapêuticas rigorosas. Estudos prospectivos randomizados de acompanhamento em larga escala são necessários para confirmar a causalidade da associação encontrada em nosso estudo.
