# Supplementary material for: O Índice de Inflamação Imune Sistêmica é um Novo Marcador na Previsão da Presença e Gravidade aa Ectasia Coronariana Isolada
Source: Arq Bras Cardiol. 2022 Dec 20;120(1):e20220056. [Article in Portuguese] doi: 10.36660/abc.20220056 (PMC9833212; doi:10.36660/abc.20220056)
Supplement: Translation

# Translation: Systemic Immune Inflammation Index is a Novel Marker in Predicting the Presence and Severity of Isolated Coronary Artery Ectasia

**Authors:** Ferhat Dindas, Emin Koyun, Erdem Turkyilmaz, Ozge Ozcan Abacioglu, Arafat Yildirim, Anil Sahin, Baris Dindar, Mustafa Dogdus, Ozkan Candan

**Affiliations:** 1 Turkey Usak University, Training and Research Hospital, Department of Cardiology, Usak – Turkey; 2 Turkey Sivas Cumhuriyet University, Department of Cardiology, Sivas – Turkey; 3 Turkey University of Health Sciences, Adana Health Practice and Research Center, Adana – Turkey

**Keywords:** Coronary Artery Disease/complications, Dilatation Pathologic, Systemic Inflammation Immune

## Abstract

**Background:**

The underlying pathology of isolated coronary artery ectasia (CE) has not been fully elucidated.

**Objective:**

We aimed to examine the relationship between the systemic immune inflammation index (Sıı), which corresponds to the multiplying of the neutrophil-to-lymphocyte ratio (NLR) and the platelet counts, and isolated CE.

**Method:**

The retrospective study population included 200 patients with isolated CE, 200 consecutive with obstructive coronary artery disease, and 200 consecutive with a normal coronary artery angiogram. A 2-sided p-value of <0.05 was considered significant.

**Results:**

Sıı, NLR, platelet-to-lymphocyte ratio (PLR), and monocyte-to-high density lipoprotein cholesterol ratio (MHR) were significantly higher in the CE group compared with the other groups (all p<0.001). In multivariate analysis, Sıı (p<0.001, OR = 1.005, 95% CI =1.004-1.005) was found to be an independent predictor of isolated CE. In Receiver Operating Characteristic curve analysis, Sıı had a higher Area Under the Curve than NLR, PLR, and MHR. Sıı value of >517.35 has 79% sensitivity, 76% specificity for the prediction of the CE [AUC: 0.832, (p<0.001)]. Sıı had a significant correlation with the number of ectatic coronary arteries and Markis classification (r:0.214 p=0.002; r:-0.195, p=0.006, respectively).

**Conclusion:**

To the best of our knowledge, this is the first study that Sıı was significantly associated with isolated CE presence and anatomical severity.

## Introduction

Coronary artery ectasia (CE) is defined as the enlargement of one or more segments of the epicardial coronary artery exceeding 1.5 times the adjacent segment. ^1^ CE is a pathological finding angiographically non-occlusive in the coronary arteries. ^2^ CE is an important clinical entity since it develops occlusive coronary artery disease (CAD) at 34%. ^3^ Many complex cellular and molecular components are involved in the pathobiological cascade of CE. Recent studies comparing CE with CAD and coronary artery aneurysms found that pathology based on inflammation is predominant. ^4 - 6^

Inflammatory biomarkers in the hematological provenance, especially the neutrophil-to-lymphocyte ratio (NLR) and platelet-to-lymphocyte ratio (PLR), have recently gained a reputation for predicting cardiovascular adverse events. ^7^ Another inflammatory and atherosclerosis-related index is the monocyte-to-high density lipoprotein cholesterol ratio (MHR). ^8^ Hu et al. developed an innovative, predictable rational marker called the systemic immune inflammatory index (Sıı) based on an ambispective cohort study. ^9^ The Sıı, which corresponds to the multiplying of the NLR and the number of platelets (PN/L), is a newly defined parameter showing systemic and local immune response. ^9^ The Sıı has been found to be associated with a major adverse cardiovascular event in elderly patients with acute myocardial infarction. ^10^ Recent studies have found a correlation between Sıı and the severity of coronary artery disease. ^11 , 12^ The Sıı includes a combination of three hematological inflammatory cells, the property that makes it more valuable than other inflammatory parameters in current studies.

Accordingly, we aimed to investigate the possible relationship between isolated CE and a new inflammation parameter, Sıı, in patients with stable or unstable angina pectoris who underwent coronary angiography (CAG).

## Methods

### Study population and Ethics

We initially included 252 patients with CE retrospectively who underwent CAG with a prediagnosis of stable and unstable CAD between January 2011 and December 2019. For the diagnosis, treatment or exclusion of CAD, CAG was performed in the presence of typical chest pain and accompanied by one or more examinations such as positive treadmill tests, abnormal myocardial perfusion scintigraphy, and abnormal coronary computed tomography angiography.

Patients with a history of percutaneous coronary intervention and coronary artery bypass grafting, presence of acute myocardial infarction with ST or Non-ST elevation, infectious, hematological, inflammatory disorders, advanced renal (estimated glomerular filtration rate < 30) and hepatic insufficiency (prolonged international normalized ratio (>1.5) with increased serum total bilirubin, alanine aminotransferase and aspartate aminotransferase level) and, diagnosed malignancy were excluded from the study. The remaining 200 consecutive patients with isolated CE, the 200 consecutive patients with obstructive CAD without coronary ectasia, and the 200 consecutive patients with angiographically normal coronary artery were matched to form three groups. Hyperlipidemia was defined as total cholesterol (TC) >200 mg/dL or low-density lipoprotein cholesterol (LDL-c) >160 mg/dL or use of statin therapy. Diabetes mellitus was defined as plasma fasting glucose >126 mg/dL or using any antidiabetic agent. Positive family history was defined by a first-degree relative before the age of 55 in men and 65 in women with CAD or sudden death. Hypertension was defined as systolic blood pressure ≥ 140 mm Hg and/or diastolic blood pressure ≥ 90 mm Hg or any reported antihypertensive medication usage. Smoking was defined as smoking for more than one pack-year. The local ethics committee approved the study protocol following the Declaration of Helsinki (2021-12/09). Due to the study’s retrospective nature, patient consent was waived by the ethics committee.

### CAG assessment

CAG of all patients included in the study was performed using the Judkins technique and 6-French catheters from the femoral or radial artery. Our cardiology clinic recorded all angiographic images in the Philips Allura Xper Percutaneous Coronary Intervention digital system. Iopromide contrast (Omnipaque; GE Healthcare) was used in all study patients. The digital angiographic images recorded in at least four cine-projections for the left coronary system and at least two cine-projections for the right coronary system were evaluated. The angiographic images were evaluated by two cardiologists blinded to the study details. CE was defined as the enlargement of any segment of any major coronary artery to at least 1.5 times the diameter of the adjacent segment without a lesion causing greater than 50% stenosis. The isolated CE group was divided into four types according to the Markis classification: diffuse ectasia in two or three coronary arteries Type I; diffuse ectasia in one coronary artery and localized ectasia in other arteries Type II; diffuse ectasia in only one coronary artery Type III; localized and segmental ectatic lesions Type IV. ^13^ The ectatic coronary arteries number was obtained by evaluating only the major coronary arteries. (LMCA, LAD, LCX, RCA). The obstructive CAD group was defined as >50% stenosis in one or more main coronary arteries and no ectasia in any coronary artery. The normal coronary artery group was defined as the absence of any CAD causing visual lumen irregularity in CAG.

### Laboratory measurements

Peripheral venous blood samples were drawn from the patients before CAG at admission. Complete blood cell parameters, blood samples were collected in 3.0 ml tubes containing 5.40 mg dry ethylenediamine tetraacetic acid (EDTA) and analyzed using an automated blood cell counter (Beckman Coulter, USA). Serum levels of triglyceride (TG), high-density lipoprotein (HDL-c), and TC were quantified with standard enzymatic methods (Abbot GmbH Co, Germany) with a fully automated analyzer (Abbott Architect c16000) with original reagents. In contrast, LDL-c concentration was determined according to the Friedewald method. C-reactive protein (CRP) concentration was measured with an automated chemistry analyzer. NLR, PLR, and MHR were calculated as absolute neutrophil count/absolute lymphocyte count, absolute platelet count/absolute lymphocyte count and absolute monocyte count/absolute HDL-c level, respectively. Sıı was calculated as absolute neutrophil count x platelet count/absolute lymphocyte count. ^14^

### Statistical analysis

All study data were analyzed with SPSS software (IBM SPSS Statistics for Windows, Version 21.0. Armonk, NY, USA, IBM Corp.). The Kolmogorov-Smirnov test was performed to confirm whether the variables are normally distributed. Continuous variables were indicated as the median and interquartile range (25th-75th percentile), and categorical variables were indicated as frequencies and percentages. The Kruskal-Wallis H test was used to compare three independent groups to analyze the variables that do not fit the normal distribution. Dunn-Bonferroni post hoc test was used for pairwise comparisons. The categorical variables were analyzed using the appropriate chi-square test. The correlation among variables was evaluated by using Spearman’s Rank correlation test. Receiver Operating Characteristic (ROC) curve analysis was used to determine the predictive role of variables.

Multivariate logistic regression analysis was employed to determine the independent variables for isolated CE. For multivariate regression, variables with a p-value of <0.1 in the univariate logistic regression analysis were identified as potential risk markers and included in the full model. The Hosmer–Lemeshow test ascertained an adequate fit for the regression model. Platelets, neutrophils, and lymphocytes were not included in the regression models to avoid multicollinearity. The odds ratios (ORs) were presented with 95% respective confidence intervals (CIs). A 2-sided p-value of <0.05 was considered significant.

## Results

Baseline clinical and angiographic characteristics of the study population are presented in Table 1 . There was no difference between the three groups regarding age, gender, LVEF, dyslipidemia, family history, hypertension, and previous medications. Smoking was higher in the ectatic coronary artery group, whereas diabetes mellitus frequency was more prevalent in the obstructive coronary artery group. The frequency of coronary arteries with ectasia in our study was as follows; right coronary artery (RCA) 62.5%, left anterior descending artery (LAD) 52%, circumflex artery (CX) 38.5%, left main coronary artery (LMCA) 6.5%. Distribution according to CE severity: Markis Type I 28.5%, Markis Type II 16.0%, Markis Type III 21.5%, Markis Type IV 34.0%. Of the four coronary artery localizations consisting of LMCA, LAD, CX, and RCA, CE was 13% in three coronary arteries, 27.5% in two coronary arteries, and 59.5% in one coronary artery. Laboratory findings of study groups are presented in Table 2 . The patients with isolated CE had significantly higher white blood cell (WBC), neutrophil counts, platelet counts, MHR, PLR, and NLR. As presented in Figure 1 , Sıı was significantly higher in the isolated CE group than in the other groups. On the other hand, the isolated CE group had significantly lower ALT levels and lymphocyte counts. CRP levels were significantly higher in the obstructive coronary artery group than in other groups. Smoking, diabetes mellitus, TG, ALT, CRP, MHR, PLR, NLR, and Sıı were evaluated by regression analysis to detect the univariate determinants of isolated CE. In the multivariate logistic regression analysis, smoking, diabetes mellitus, MHR, PLR, NLR, and Sıı were independent and significant predictors of isolated CE ( Table 3 ). In the ROC curve analysis to estimate the isolated CE, Sıı has the highest AUC with a cut-off value of 517.35 ( Figure 2 ). Sıı significantly negatively correlated with the Markis classification ( Figure 3 ). Moreover, Sıı significantly correlated with the number of ectatic coronary arteries ( Figure 4 ).

**Table 1:** Comparison of baseline clinical and angiographic characteristics of groups

Variables	Ectatic coronary artery group (n=200)	Obstructive coronary artery group (n=200)	Normal coronary artery group (n=200)	p
Age, years	58 (52 - 65)	59 (52 - 66)	59 (52 - 63)	0.348
LVEF, (%)	52 (52 - 57)	52 (52 - 55)	52 (51 - 55)	0.918
Male gender, n (%)	143 (71.5)	151 (75.5)	139 (69.5)	0.395
Dyslipidemia, n (%)	64 (32.0)	77 (38.5)	61 (30.5)	0.198
Family history, n (%)	82 (41.0)	88 (44.0)	76 (38.0)	0.475
Smoking, n (%)	86 (43.0)	70 (35.2)	48 (24.0)	<0.001
Hypertension, n (%)	110 (55.0)	103 (51.5)	97 (48.5)	0.428
Diabetes mellitus, n (%)	57 (28.5)	89 (44.5)	65 (32.8)	0.002
**Medications, n(%)**				
ACE-ARB	88 (44.0)	97 (48.5)	84 (42.0)	0.408
CCB	45 (22.5)	54 (27.0)	43 (21.5)	0.387
APT	36 (18.0)	38 (19.0)	32 (16.0)	0.726
BB	61 (30.5)	62 (31.0)	47 (23.5)	0.177
Statin therapy	71 (35.5)	76 (38.0)	62 (31.0)	0.330
**Coronary artery, n(%)**				
LMCA	13 ( 6.5 )			
LAD	104 ( 52.0 )			
CX	77 ( 38.5 )			
RCA	125 ( 62.5 )			
**Markis classification, n(%)**				
Type I	57 ( 28.5 )			
Type II	32 ( 16.0 )			
Type III	43 ( 21.5 )			
Type IV	68 ( 34.0 )			
**Number of ectatic coronary arteries, n(%)**				
1	119 ( 59.5 )			
2	55 ( 27.5 )			
3	26 ( 13.0 )			

*ACE: angiotensin-converting enzyme inhibitor; APT: antiplatelet therapy; ARB: angiotensin 2 receptor blockers; BB: beta blocker; CCB: calcium channel blocker; CX: circumflex artery; LVEF: left ventricular ejection fraction; LAD: left anterior descending artery; LMCA: left main coronary artery; RCA: right coronary artery.*

**Table 2:** Comparison of laboratory parameters of study groups

Variables	Ectatic coronary artery group (n=200)	Obstructive coronary artery group (n=200)	Normal coronary artery group (n=200)	p
BUN, mg/dL	17 (13 - 21)	16 (13 - 21)	16 (13 – 19.6)	0.535
Creatinine, mg/dL	0.9 (0.7 - 1.3)	0.97 (0.81 - 1.18)	0.95 (0.80 - 1.18)	0.600
CRP, mg/L	3.4 (1.93 – 7.58) ^ab^	2.25 (1.3 – 9.30) ^a^	4.82 (3.0 – 6.0) ^b^	**0.001**
ALT, IU/L	20 (15 - 30) ^a^	24 (18 – 33.8) ^b^	23.5 (17 - 32) ^b^	**0.005**
Hemoglobin, g/dL	14.8 (13.4 – 15.6)	14.2 (13.2 - 15.5)	14.3 (13.1 – 15.4)	0.190
RDW, %	11.85 (11.5 - 12.6)	11.9 (11.5 - 12.9)	12.1 (11.5 - 13.1)	0.212
WBC, x10 ^9^ /L	9.16 (7.83 – 10.86) ^a^	8.72 (7.1 – 10.8) ^a^	7.79 (6.64 – 9.25) ^b^	**<0.001**
Platelet, x10 ^9^ /L	279 (235 – 321.7) ^a^	222.5 (192.3 – 254.5) ^b^	226 (195 - 261) ^b^	**<0.001**
MCV (fL)	87.05 (80.5 – 90.1)	87.2 (84.9 - 90.1)	87.1 (80.4 - 90.1)	0.552
MPV (fL)	7.6 (7.1 - 8.3)	7.4 (7.0 - 8.2)	7.7 (6.9 - 8.3)	0.327
Neutrophil, x10 ^9^ /L	5.7 (4.63 – 6.99) ^a^	4.97 (3.82 – 6.48) ^b^	3.58 (2.19 – 5.05) ^c^	**<0.001**
Monocyte, x10 ^9^ /L	0.53 (0.41 - 0.73) ^a^	0.49 (0.38 - 0.61) ^b^	0.51 (0.39 - 0.61) ^b^	**0.003**
Lymphocyte, x10 ^9^ /L	2.21 (1.75 - 2.77) ^a^	2.69 (2.04 – 3.22) ^b^	2.7 (2.17 – 3.40) ^b^	**<0.001**
TG mg/dL	126 (91 - 182)	141 (103 – 200.7)	130 (94 - 192)	0.081
TC, mg/dL	175 (143 - 203)	185 (157 – 214.3)	180 (150 - 208)	0.222
LDL-c, mg/dL	104 (77.7 - 125)	104.3 (78 - 134)	98 (75 - 123)	0.529
HDL-c, mg/dL	37 (31 - 45)	38 (32.2 - 46)	36 (31 - 44)	0.256
MHR	0.01 (0.01 - 0.02) ^a^	0.01 (0.01 - 0.02) ^b^	0.01 (0.1 - 0.02) ^ab^	**<0.001**
NLR	2.59 (1.94 - 3.40) ^a^	1.83 (1.41 - 2.76) ^b^	1.3 (0.74 - 1.98) ^c^	**<0.001**
PLR	124.2 (101.3 - 163.0) ^a^	82.6 (66.4 - 106.8) ^b^	85.4 (60.9 – 103.0) ^b^	**<0.001**
Sıı	720.1 (532.9 - 991.8) ^a^	427.6 (307.5 – 597.8) ^b^	278.1 (172.0 - 436.1) ^c^	**<0.001**

*ALT: alanine aminotransferase; BUN: blood urea nitrogen; CRP: C-reactive protein; HDL-c: t to HDL-c ratio; NLR: neutrophil to lymphocyte ratio; MCV: mean corpuscular volume; MPV: mean platelet volume; LDL-c: low-density lipoprotein cholesterol; PLR: platelet to lymphocyte ratio; RDW: red cell distribution width; Sıı: systemic immune-inflammation index; TC: total cholesterol; TG: triglyceride; WBC: white blood cell; MHR: monocyte-to-high density lipoprotein cholesterol ratio. Variables are expressed as median (25th–75th). Similar letters in the same line show the similarity between groups; different letters show the difference between groups.*

**Figure 1:** Sıı values of groups.

**Table 3:** Predictors of isolated CE by multivariate logistic regression analysis

Variables	Univariate Analysis		Multivariate Analysis	

	p	OR (95% CI)	p	Adjusted OR (95% CI)
Smoking	0.001	1.796 (1.262-2.558)	<0.001	1.824 (1.189-2.803)
Diabetes mellitus	0.014	0.632 (0.435-0.908)	<0.001	0.644 (0.412-0.998)
TG	0.256	0.999 (0.997-1.001)		
ALT	0.320	0.996 (0.986-1.004)		
CRP	0.233	1.016 (0.99-1.042)		
MHR	<0.001	1.062 (1.036-1.089)	<0.001	1.039 (1.007-1.073)
PLR	<0.001	1.026 (1.021-1.032)	<0.001	1.027 (1.022-1.033)
NLR	<0.001	2.02 (1.717-2.4)	<0.001	2.024 (1.708-2.423)
Sıı	<0.001	1.005 (1.004-1.005)	<0.001	1.004 (1.004-1.005)

*CE: coronary artery ectasia; CI: confidence interval; OR: odds ratio; p: p-value. ALT: alanine aminotransferase; CRP: C-reactive protein; NLR: neutrophil to lymphocyte ratio; PLR: platelet to lymphocyte ratio; Sıı: systemic immune-inflammation index; TG: triglyceride; MHR: monocyte-to-high density lipoprotein cholesterol ratio.*

**Figure 2:** ROC curve analysis of Sıı, NLR, PLR, and MHR for predicting isolated CE. CI: confidence interval; NLR: neutrophil to lymphocyte ratio; PLR: platelet to lymphocyte ratio; Sıı: systemic immune-inflammation index; MHR: monocyte-to-high density lipoprotein cholesterol ratio.

**Figure 3:** Correlation of Sıı with the Markis classification.

**Figure 4:** Correlation of Sıı with the number of the ectatic coronary artery.

## Discussion

We aimed to investigate the relationship between isolated CE and Sıı, a novel marker including neutrophil, platelet, and lymphocyte counts. Our study showed that isolated CE patients had significantly higher Sıı values than patients with obstructive and normal coronary arteries. In particular, the severity of isolated CE and its extent in the coronary arteries are independently associated with an increase in Sıı, reflecting inflammatory activation.

Dilated coronary segments, impaired antegrade coronary dye filling, localized dye deposition with stasis, and retrograde leak phenomenon are CE’s main coronary angiographic features. ^15^ The reported incidence of CE ranges from 0.3% to 10%, depending on the number of patients undergoing CAG. ^16 , 17^ In a cross-sectional study, it was found that the frequency of cardiovascular events increased in the presence of CE accompanying CAD. ^18^ In another recent study, CE’s presence affects future cardiovascular mortality in patients with acute myocardial infarction. ^19^ Increasing detection of coronary artery ectasia, showing its relationship with cardiovascular mortality, is one of the predisposing factors of acute myocardial infarction and associated with microvascular ischemia in the absence of concomitant obstructive lesion, has increased its clinical importance in the last decade. ^15 , 18 - 20^ The coronary artery most affected by ectasia is RCA, followed by LAD and CX, and the least affected coronary artery is LMCA. ^6^ CE has demographic characteristics of younger men compared to obstructive CAD patients. ^21^ In our study, there was no significant demographic difference between all groups, and patients with isolated CE were similar to patients with obstructive CAD in terms of cardiovascular event risk factors. Consistent with the literature, in our study, there was a linear relationship between smoking, hypertension and hyperlipidemia in patients with CE, while an inverse relationship was found with diabetes mellitus. ^2^ This inverse relationship is due to diabetes mellitus impairing compensatory arterial dilation by promoting the atherosclerotic process to negative arterial wall remodeling. ^4^ The title of the atherosclerotic process in the pathogenesis of CE, which is considered a type of CAD, remains unclear. ^5^ Based on results from these previous publications comparing CE and coronary artery aneurysms, inflammatory activity is more obvious in CE. ^22^ For these reasons, although the pathogenesis of CE has not been fully elucidated, inflammatory and other probable mechanisms rather than the atherosclerotic process have become more highlighted topics. ^4 , 5^

There are several studies on the relationship between CE and inflammation. Demir et al. ^23^ showed that high serum high sensitive-CRP, uric acid levels and lower serum bilirubin levels, which are indicators of the inflammatory response, are associated with the presence of ectasia. ^23^ Turan et al. ^24^ illustrated a correlation between endocan levels indicating endothelial dysfunction and inflammatory process and CE. ^24^ Finkelstein et al. ^25^ showed that circulatory matrix metalloproteinase imbalance, which is correlated with inflammatory markers, is associated with CE formation. ^25^ Jun Li et al. ^5^ illustrated that CE was associated with interleukin-6, WBC, neutrophil, and monocyte counts and claimed that the chronic inflammatory process is involved in the CE. ^5^ Ashraf et al. ^26^ found a correlation between the adipocyte hormone visfatin, which plays a key role in delayed neutrophil apoptosis, and the severity of CE. ^26^ Adiloglu et al. ^27^ showed that cell surface adhesion molecules, which are necessary for the initiation of inflammation in CE, are more widespread in the endothelium. ^27^ In addition, several studies have found that NLR, PLR and MHR, which are traditional inflammatory parameters, are independent predictors of CE and are correlated with the number and severity of ectatic vessels. ^28 - 30^ Consistently with this, NLR, PLR and MHR were independent predictors of isolated CE in our study. Although CRP was higher in the isolated CE group than in the other groups, we could not detect it as an independent predictor. This result may be due to our study’s absence of high sensitive-CRP values.

Sıı is a new hematological inflammatory marker that brings together neutrophils, platelets, and lymphocytes, reflecting inflammation and immune balance. Sıı was first defined in the oncology field. The excess systemic inflammatory index has been associated with circulating cancer cell count, poor prognosis, and shorter survival time in cancer patients. ^9 , 14^ In lately cancer studies, the prognostic role of Sıı was found to be more potent than NLR and PLR. ^31^ Also, Sıı as a prognostic marker has been demonstrated in cardiovascular disease patients. ^10 - 12^ There is increasing evidence that neutrophils and neutrophil-derived products participate in CE. ^32^ In the pathobiological process of CE, there is an increase in cell surface adhesive molecules. ^26^ Subsequently, extracellular matrix degranulation occurs due to adhesin molecules providing monocyte transmigration and spreading neutrophil-derived serine proteinase. ^26 , 33 , 34^ Previous studies reported that platelets, chemokines, and cytokines increase the migration of progenitor cells to the inflammatory area that develops after endothelial injury. ^5 , 33^ Platelets play important roles in inflammation and thrombosis. Activated platelet aggregation is known to be among the main cause of cardiovascular complications. ^35^ The platelet activation marker, mean platelet volume, has recently been shown to be of prognostic value in major adverse cardiac events in patients with isolated CE. ^36^ As a result of previous publication, plasma P-selectin and platelet factor-4 levels, which are thought to increase platelet activation in patients with isolated CE, were higher than in the control group. ^37^ In contrast to neutrophile and platelet, the immune modulation function of lymphocytes in inflammation is explained by increased lymphocyte apoptosis, down-regulation of lymphocyte proliferation and differentiation, and decreased lymphocyte counts. ^28^ In addition, it is known that neutrophilic and lymphopenic leukocytosis are poor prognostic markers of several main cardiovascular diseases. ^28^ In light of reported studies, we hypothesized that the level of Sıı, which is known to be associated with inflammation and immune system activation, may predict patients with CE. ROC curve analyses in our study showed that Sıı had a higher AUC compared to NLR, PLR and, MHR; this led us to conclude that Sıı predicts isolated CE patients better than NLR, PLR and, MHR. Also, Sıı emerged as an independent predictor of isolated CE in the multivariate regression models. However, there was a powerless correlation between Sıı and the anatomical severity of isolated CE. According to these results, among the conventional hematological indices that can be obtained non-invasively from the complete blood count results, Sıı is more applicable in predicting patients with isolated CE. In addition, Sıı may be an interesting marker that deserves investigation in diverse cardiac pathologies.

There are a few limitations in our study that should be mentioned. It is a single-center retrospective small sample study; the results lack long-term follow-up and outcome measures. Besides, the study did not include long-term follow-up and outcome measures showing prognostic efficacy. CRP-to-albumin ratio, another important inflammatory parameter, was not evaluated due to the lack of albumin value in laboratory results before CAG. In addition, although patient angiographic assessments were determined by two cardiologists blinded to the study data, the possibility of bias cannot be completely excluded because inter or intra-observer variability was not calculated. Even though the study includes data on the number of ectatic coronary arteries and Markis classification, Sıı does not directly reflect isolated CE’s anatomical and inflammatory importance since techniques to investigate systemic immune vasculitides in the etiology were not used.

## Conclusion

In conclusion, CE is associated with increased Sıı and suggests the presence of inflammatory processes in patients with isolated CE. We think Sıı may be a more effective index than other hematological inflammatory parameters in differentiating isolated CE patients who will need rigorous therapeutic strategies. Large-scale follow-up prospective randomized studies are needed to confirm the causality of the association found in our study.
